# Technical Issues of [^18^F]FET-PET Imaging for Radiation Therapy Planning in Malignant Glioma Patients – A Review

**DOI:** 10.3389/fonc.2012.00130

**Published:** 2012-10-04

**Authors:** F. Walter, C. la Fougère, C. Belka, M. Niyazi

**Affiliations:** ^1^Department of Radiation Oncology, University of MunichMunich, Germany; ^2^Department of Nuclear Medicine, University of MunichMunich, Germany

## Introduction

Radiotherapy either alone or in combination with temozolomide is a key element for the treatment of high-grade brain tumors at primary diagnosis and even at recurrence of disease (Stupp et al., 2009; Niyazi et al., 2011b). Nevertheless the clinical course of primary malignant brain tumors remains dismal, since early local failure is common. At least in part local failure is related to a diffuse tumor growth and difficulties in visualizing the relevant borders of the tumor contaminated area. Advances in radiation therapy technique such as 3D-conformal radiation, stereotactic radiotherapy (Combs et al., 2005), and intensity modulated radiotherapy (IMRT; Veldeman et al., 2008; Jin et al., 2011) as well as improved image-guidance radiotherapy (IGRT; Minniti et al., 2010; Wilbert et al., 2010) provide the ability to deliver radiation dose with increasing precision. In order to take full advantage of these technological advances there is a need for precise tumor delineation and target definition.

This subsequently demands for precise imaging. Besides conventional radiotherapy planning being still based on computed tomography (CT) due to dose calculation algorithms additional magnetic resonance imaging (MRI) has become current standard-of-care with a high soft tissue contrast (for planning purposes and during follow-up Gladwish et al., 2011). Current and future research in this field are focused on the meaning of special or new sequence techniques (Laprie, 2009; Stall et al., 2010). Positron emission tomography (PET) has become a further option in recent years (Grosu et al., 2005a).

## Amino Acid PET Imaging

Concerning high-grade brain tumors standard imaging modalities such as CT or MRI are by far not tumor-specific. In addition they are only of limited value when a differentiation between viable tumor and treatment related changes such as edema, disturbance of the blood brain barrier, or necrosis is needed. In clinical reality, morphological changes associated with clinical signs indicative of tumor progression may occur without real tumor growth as treatment consequence (pseudo-progression). This phenomenon is seen in up to 30% of patients (Wen et al., 2010) and patients with pseudo-progression recover spontaneously without further treatment (Brandsma et al., 2008). In this context, diagnosis of a true recurrence remains often challenging.

While conventional imaging modalities provide only anatomical information on tumor size and localization, PET as a molecular imaging modality provides additional information on the metabolic activity of the tumor (Weber, 2006).

Several radio-labeled PET tracer molecules have been used for imaging of patients with brain tumors. These tracers are used to visualize glucose metabolism, proliferation, protein synthesis, hypoxia, and the expression of amino acid transporters (La Fougere et al., 2011). The most widely used PET tracer ^18^F-fluorodeoxyglucose ([^18^F]FDG) however is of limited use for brain tumor imaging due to the high physiological glucose metabolism of the cerebral cortex.

Amino acid tracers have more favorable imaging characteristics for malignant disease of the brain due to a low background uptake in normal brain tissue. In addition, the uptake of amino acids is frequently up regulated in high-grade gliomas (Kobayashi et al., 2008). Therefore amino acid PET produces images with a high tumor-to-background contrast in patients with high and also with low grade brain tumors (Fueger et al., 2010).

Several amino acid tracers have been clinically applied for imaging of patients suffering from gliomas in both at primary diagnosis as well as recurrent disease. A couple of studies report the assessment of treatment response with amino acid imaging and an impact on the management of these patients could be shown (Walter et al., 2012). Among these tracers ^11^C-methionine ([^11^C]MET), 3,4-dihydroxy-6-^18^F-fluoro-l-phenylalanine ([^18^F]DOPA), and ^18^F-fluoroethyl-l-tyrosine ([^18^F]FET) are the most widely used tracers. Available data suggest that different amino acid tracers have comparable imaging characteristics in brain tumors (Becherer et al., 2003). In clinical practice however, ^18^F-labeled PET molecules are advantageous to those that are ^11^C-labeled due to the longer physical half-life of 110 min vs. 20 min which averts the need of an on-site cyclotron.

## Target Volume Delineation

The use of molecular imaging in treatment planning of brain tumor patients is focused on tumor volume delineation. There has been an increased use of PET imaging for radiotherapy treatment planning in other cancer types as several studies reported the benefit of [^18^F]FDG-PET imaging for target volume selection for radiotherapy (De Ruysscher and Kirsch, 2010). Most frequently PET information was used for staging and lymph node detection (Tsai et al., 2010).

The accurate tumor volume definition is crucial to prevent geographical misses and to minimize toxicity to normal brain tissue. Gadolinium enhanced MRI T_1_-weighted is the standard imaging technique for gross tumor volume (GTV) definition in patients with malignant brain tumors. Reported data however suggest that a biological target volume (BTV) defined by amino acid PET in resected patients with brain tumors differs from GTV defined by MRI (Grosu et al., 2005c).

Both imaging techniques must be regarded as complementary (Weber et al., 2009). Biologically image-guided target volume delineation of clinical target volume (CTV) therefore includes GTV definition with anatomical imaging and BTV definition based on molecular imaging. Available data suggest that the use of additional [^18^F]FET-PET information to conventional imaging for radiation treatment planning might improve tumor volume delineation in patients with macroscopic tumor (Niyazi et al., 2011a). It was shown that the GTV based on Gadolinium enhancement and a BTV defined by [^18^F]FET uptake differed significantly in size and geometrical extension in a majority of patients (Weber et al., 2008; Niyazi et al., 2011a).

## Contouring Algorithms

The direct use of PET data for contouring purposes is currently under investigation. At present there are no established objective tools for this purpose. A considerable limitation of PET imaging compared to conventional imaging techniques is the limited spatial resolution of about 5 mm which does not allow for exact definition of tumor borders (Piroth et al., 2009). Thus contouring of the tumor volume needs to be performed using conventional and metabolic imaging side-by-side. Despite all given problems it has been shown clearly that the use of PET scans for tumor volume contouring in lung cancer patients significantly reduced inter-observer variability due to the high tumor-to-background contrast (De Ruysscher et al., 2012). Comparable data are to our knowledge not available for [^18^F]FET and brain tumors at present – Weber et al. (2008) solely compared the interrater reliability for BTV delineation which was excellent due to the use of [^18^F]FET.

Several approaches for PET-based target volume contouring are feasible and utilized in various studies. Visual interpretation of PET images side-by-side with conventional imaging is currently most commonly executed. The GTV is defined by uptake clearly above the background activity. This method is relatively prone to inter-observer variation and standardization is not readily achievable. Therefore more objective methods of tumor volume contouring are under investigation.

The most widely used parameter for PET imaging quantification is the maximum standardized uptake value (SUV_max_). It is calculated for selected regions of interest (ROIs) and represents the voxel of maximum uptake within the ROI. However the reproducibility of the SUV_max_ in clinical practice is controversial (Guha et al., 2008) since it depends on several factors such as patient preparation, uptake-kinetics, imaging time or imaging acquisition, and reconstruction method as well as the size of the lesion due to partial volume effects. Several approaches of contouring based on SUVs have been applied. Segmentation methods using either absolute SUV_max_, fixed percentages of the maximum uptake, or SUV_max_/background ratio have been proposed (Schinagl et al., 2007). It has been suggested that the use of a SUV_max_/background ratio is less dependent on external factors as absolute SUV_max_ (Pauleit et al., 2005). A threshold of SUV_max_/background ratio in the range of 1.6–2.1 (Dunet et al., 2012; Piroth et al., 2012) has been applied in several studies.

## Dose Escalation by Contours and Numbers

Malignant gliomas are relatively radio-resistant tumors. Therefore high radiation dose is needed for sufficient control of this disease. However, the application of radiation dose is restricted by the neighboring organs at risk (OAR). The introduction of IMRT allows for heterogeneous delivery of radiation dose (Ling et al., 2000) while sparing of normal tissue becomes easier.

As the addition of biological imaging such as [^18^F]FET uptake in brain tumors displays the metabolic activity within the tumor volume this three dimensional information can be used to modify the dose distribution within the target volume to accomplish a higher radiation dose to intra-tumoral regions of relatively higher radio-resistance (Madani et al., 2007).

In this regard, dose painting by contours (DPBC) is a method of biological image-guided radiotherapy that defines sub-volumes of areas with high metabolic tumor activity inside a conventionally CT based target volume (Thorwarth et al., 2010). An escalation of radiation dose to these defined sub-volumes is applied. Using DPBC the radiation dose prescribed to the defined sub-volumes is homogeneously delivered and may e.g., be applied in prostate cancer (Niyazi et al., 2010; Wurschmidt et al., 2011; Chang et al., 2012), lung cancer (Meijer et al., 2011), and many other cancer types.

Another method of specific dose escalation in a PET-derived area within the tumor is dose painting by numbers (DPBN; Bentzen, 2005; Thorwarth et al., 2007a). DPBN is designed to deliver the dose more effectively than an additional uniform boost to the PET-positive area. The intensity of tracer uptake is measured voxel-wise and an inhomogeneous radiation dose is delivered voxel-by-voxel. The theoretical feasibility of a [^18^F]FET-PET based DPBN approach in brain tumors was shown by Rickhey et al. (2008) and later applied on proton therapy (Rickhey et al., 2010).

A recent prospective study confirmed that dose escalation with [^18^F]FET-PET planning is feasible (Piroth et al., 2012). However, this study could not show a survival benefit for these patients.

It has to be elucidated whether dynamic analyses add substantial benefit to target volume definition as its role in defining more malignant parts of the tumor remains open (La Fougere et al., 2011).

One further DPBN model system is actually head-and-neck cancer where hypoxia was quantified with a simple imaging technique like [^18^F]-fluoromisonidazole (FMISO) PET. A tumor control probability model was developed based on repeated FMISO PET scans during radiotherapy. The model combined the local perfusion efficiency and the degree of hypoxia to estimate reoxygenation time (Thorwarth et al., 2007b).

Concerning glioblastoma, volume, and intensity of hypoxia before radiotherapy seem to be strongly associated with poorer time-to-progression and survival (Spence et al., 2008). It was shown that complementary use of ^11^C-methionine (MET) and FMISO to Gadolinium-enhanced MRI may improve the understanding of tumor biology and lead to more efficient delineation of tumor volume and an improved treatment strategy (Kawai et al., 2010) but further studies are lacking up to now.

## Future Perspectives

The role of amino acid PET for imaging of brain tumors is well established and the applicability of molecular imaging for radiotherapy planning has been shown. There is a growing body of knowledge on the use of molecular imaging with [^18^F]FET-PET in radiotherapy planning of brain tumor patients. Available data suggest that [^18^F]FET-PET imaging can contribute valuable information for tumor volume delineation. At present the development of semi-automated tumor delineation tools using segmentation methods based on PET imaging are under investigation (Cheebsumon et al., 2011) and it has been shown that dose escalation based on PET imaging is feasible (Piroth et al., 2012).

In a single-center study on 44 patients with recurrent glioma it could be shown that patients planned with amino acid PET had a significantly longer survival than those with conventional imaging for radiation treatment planning only (Grosu et al., 2005b) reinforcing the potential of [^18^F]FET-PET imaging for radiation therapy planning.

However, there is a need for standardization of the [^18^F]FET-PET scanning technique to allow for inter-institutional comparability. Further research preferentially based on randomized prospective trials is needed to examine the relevance of dynamic [^18^F]FET-PET analysis (La Fougere et al., 2011).

## References

[B1] Becherer A. Karanikas G. Szabo M. Zettinig G. Asenbaum S. Marosi C. (2003). Brain tumour imaging with PET: a comparison between [18F]fluorodopa and [11C]methionine. Eur. J. Nucl. Med. Mol. Imaging 30, 1561–1567 10.1007/s00259-003-1259-1 14579097

[B2] Bentzen S. M. (2005). Theragnostic imaging for radiation oncology: dose-painting by numbers. Lancet Oncol. 6, 112–117 10.1016/S1470-2045(05)01737-7 15683820

[B3] Brandsma D. Stalpers L. Taal W. Sminia P. Van Den Bent M. J. (2008). Clinical features, mechanisms, and management of pseudoprogression in malignant gliomas. Lancet Oncol. 9, 453–461 10.1016/S1470-2045(08)70125-6 18452856

[B4] Chang J. H. Lim Joon D. Lee S. T. Gong S. J. Anderson N. J. Scott A. M. (2012). Intensity modulated radiation therapy dose painting for localized prostate cancer using (11)c-choline positron emission tomography scans. Int. J. Radiat. Oncol. Biol. Phys. 83, e691–e696 10.1016/j.ijrobp.2011.10.050 22658218

[B5] Cheebsumon P. Yaqub M. Van Velden F. H. Hoekstra O. S. Lammertsma A. A. Boellaard R. (2011). Impact of [(1)(8)F]FDG PET imaging parameters on automatic tumour delineation: need for improved tumour delineation methodology. Eur. J. Nucl. Med. Mol. Imaging 38, 2136–2144 10.1007/s00259-011-1899-5 21858528 PMC3228515

[B6] Combs S. E. Widmer V. Thilmann C. Hof H. Debus J. Schulz-Ertner D. (2005). Stereotactic radiosurgery (SRS): treatment option for recurrent glioblastoma multiforme (GBM). Cancer 104, 2168–2173 10.1002/cncr.21429 16220556

[B7] De Ruysscher D. Kirsch C. M. (2010). PET scans in radiotherapy planning of lung cancer. Radiother. Oncol. 96, 335–338 10.1016/j.radonc.2010.07.002 20656364

[B8] De Ruysscher D. Nestle U. Jeraj R. Macmanus M. (2012). PET scans in radiotherapy planning of lung cancer. Lung Cancer 75, 141–145 10.1016/j.lungcan.2011.07.018 21920625

[B9] Dunet V. Rossier C. Buck A. Stupp R. Prior J. O. (2012). Performance of 18F-fluoro-ethyl-tyrosine (18F-FET) PET for the differential diagnosis of primary brain tumor: a systematic review and metaanalysis. J. Nucl. Med. 53, 207–214 10.2967/jnumed.111.096859 22302961

[B10] Fueger B. J. Czernin J. Cloughesy T. Silverman D. H. Geist C. L. Walter M. A. (2010). Correlation of 6-18F-fluoro-L-dopa PET uptake with proliferation and tumor grade in newly diagnosed and recurrent gliomas. J. Nucl. Med. 51, 1532–1538 10.2967/jnumed.110.078592 20847166

[B11] Gladwish A. Koh E. S. Hoisak J. Lockwood G. Millar B. A. Mason W. (2011). Evaluation of early imaging response criteria in glioblastoma multiforme. Radiat. Oncol. 6, 121 10.1186/1748-717X-6-121 21943399 PMC3189120

[B12] Grosu A. L. Piert M. Weber W. A. Jeremic B. Picchio M. Schratzenstaller U. (2005a). Positron emission tomography for radiation treatment planning. Strahlenther. Onkol. 181, 483–499 10.1007/s00066-005-1422-7 16044216

[B13] Grosu A. L. Weber W. A. Franz M. Stark S. Piert M. Thamm R. (2005b). Reirradiation of recurrent high-grade gliomas using amino acid PET (SPECT)/CT/MRI image fusion to determine gross tumor volume for stereotactic fractionated radiotherapy. Int. J. Radiat. Oncol. Biol. Phys. 63, 511–519 10.1016/j.ijrobp.2005.07.867 16168843

[B14] Grosu A. L. Weber W. A. Riedel E. Jeremic B. Nieder C. Franz M. (2005c). L-(methyl-11C) methionine positron emission tomography for target delineation in resected high-grade gliomas before radiotherapy. Int. J. Radiat. Oncol. Biol. Phys. 63, 64–74 10.1016/j.ijrobp.2005.07.225 16111573

[B15] Guha C. Alfieri A. Blaufox M. D. Kalnicki S. (2008). Tumor biology-guided radiotherapy treatment planning: gross tumor volume versus functional tumor volume. Semin. Nucl. Med. 38, 105–113 10.1053/j.semnuclmed.2007.12.001 18243845

[B16] Jin J. Y. Wen N. Ren L. Glide-Hurst C. Chetty I. J. (2011). Advances in treatment techniques: arc-based and other intensity modulated therapies. Cancer J. 17, 166–176 10.1097/PPO.0b013e31821f8318 21610470

[B17] Kawai N. Maeda Y. Kudomi N. Miyake K. Okada M. Yamamoto Y. (2010). Correlation of biological aggressiveness assessed by 11C-methionine PET and hypoxic burden assessed by 18F-fluoromisonidazole PET in newly diagnosed glioblastoma. Eur. J. Nucl. Med. Mol. Imaging 38, 441–450 10.1007/s00259-010-1645-4 21072512

[B18] Kobayashi K. Ohnishi A. Promsuk J. Shimizu S. Kanai Y. Shiokawa Y. (2008). Enhanced tumor growth elicited by L-type amino acid transporter 1 in human malignant glioma cells. Neurosurgery 62, 493–503; discussion 503–494. 10.1227/01.neu.0000316018.51292.19 18382329

[B19] La Fougere C. Suchorska B. Bartenstein P. Kreth F. W. Tonn J. C. (2011). Molecular imaging of gliomas with PET: opportunities and limitations. Neurooncology 13, 806–819 10.1093/neuonc/nor054 PMC3145468 21757446

[B20] Laprie A. (2009). Proton magnetic resonance spectroscopic imaging and other types of metabolic imaging for radiotherapy planning in adult and pediatric high-grade gliomas. Cancer Radiother. 13, 556–561 10.1016/j.canrad.2009.07.003 19766525

[B21] Ling C. C. Humm J. Larson S. Amols H. Fuks Z. Leibel S. (2000). Towards multidimensional radiotherapy (MD-CRT): biological imaging and biological conformality. Int. J. Radiat. Oncol. Biol. Phys. 47, 551–560 10.1016/S0360-3016(00)00467-3 10837935

[B22] Madani I. Duthoy W. Derie C. De Gersem W. Boterberg T. Saerens M. (2007). Positron emission tomography-guided, focal-dose escalation using intensity-modulated radiotherapy for head and neck cancer. Int. J. Radiat. Oncol. Biol. Phys. 68, 126–135 10.1016/j.ijrobp.2006.12.070 17448871

[B23] Meijer G. Steenhuijsen J. Bal M. De Jaeger K. Schuring D. Theuws J. (2011). Dose painting by contours versus dose painting by numbers for stage II/III lung cancer: practical implications of using a broad or sharp brush. Radiother. Oncol. 100, 396–401 10.1016/j.radonc.2011.08.048 21955663

[B24] Minniti G. Valeriani M. Clarke E. D'arienzo M. Ciotti M. Montagnoli R. (2010). Fractionated stereotactic radiotherapy for skull base tumors: analysis of treatment accuracy using a stereotactic mask fixation system. Radiat. Oncol. 5, 1 10.1186/1748-717X-5-1 20070901 PMC2823752

[B25] Niyazi M. Bartenstein P. Belka C. Ganswindt U. (2010). Choline PET based dose-painting in prostate cancer – modelling of dose effects. Radiat. Oncol. 5, 23 10.1186/1748-717X-5-23 20298546 PMC2848061

[B26] Niyazi M. Geisler J. Siefert A. Schwarz S. B. Ganswindt U. Garny S. (2011a). FET-PET for malignant glioma treatment planning. Radiother. Oncol. 99, 44–48 10.1016/S0167-8140(11)71114-8 21458093

[B27] Niyazi M. Siefert A. Schwarz S. B. Ganswindt U. Kreth F. W. Tonn J. C. (2011b). Therapeutic options for recurrent malignant glioma. Radiother. Oncol. 98, 1–14 10.1016/j.radonc.2010.11.006 21159396

[B28] Pauleit D. Floeth F. Hamacher K. Riemenschneider M. J. Reifenberger G. Muller H. W. (2005). O-(2-[18F]fluoroethyl)-L-tyrosine PET combined with MRI improves the diagnostic assessment of cerebral gliomas. Brain 128, 678–687 10.1093/brain/awh399 15689365

[B29] Piroth M. D. Pinkawa M. Holy R. Klotz J. Schaar S. Stoffels G. (2012). Integrated boost IMRT with FET-PET-adapted local dose escalation in glioblastomas. Results of a prospective phase II study. Strahlenther. Onkol. 188, 334–339 10.1007/s00066-011-0060-5 22349712

[B30] Piroth M. D. Pinkawa M. Holy R. Stoffels G. Demirel C. Attieh C. (2009). Integrated-boost IMRT or 3-D-CRT using FET-PET based auto-contoured target volume delineation for glioblastoma multiforme – a dosimetric comparison. Radiat. Oncol. 4, 57 10.1186/1748-717X-4-57 19930657 PMC2787527

[B31] Rickhey M. Koelbl O. Eilles C. Bogner L. (2008). A biologically adapted dose-escalation approach, demonstrated for 18F-FET-PET in brain tumors. Strahlenther. Onkol. 184, 536–542 10.1007/s00066-008-1883-6 19016044

[B32] Rickhey M. Moravek Z. Eilles C. Koelbl O. Bogner L. (2010). 18F-FET-PET-based dose painting by numbers with protons. Strahlenther. Onkol. 186, 320–326 10.1007/s00066-010-2014-8 20559789

[B33] Schinagl D. A. Vogel W. V. Hoffmann A. L. Van Dalen J. A. Oyen W. J. Kaanders J. H. (2007). Comparison of five segmentation tools for 18F-fluoro-deoxy-glucose-positron emission tomography-based target volume definition in head and neck cancer. Int. J. Radiat. Oncol. Biol. Phys. 69, 1282–1289 10.1016/j.ijrobp.2007.07.2333 17967318

[B34] Spence A. M. Muzi M. Swanson K. R. O'sullivan F. Rockhill J. K. Rajendran J. G. (2008). Regional hypoxia in glioblastoma multiforme quantified with [18F]fluoromisonidazole positron emission tomography before radiotherapy: correlation with time to progression and survival. Clin. Cancer Res. 14, 2623–2630 10.1158/1078-0432.CCR-07-4995 18451225 PMC4415875

[B35] Stall B. Zach L. Ning H. Ondos J. Arora B. Shankavaram U. (2010). Comparison of T2 and FLAIR imaging for target delineation in high grade gliomas. Radiat. Oncol. 5, 5 10.1186/1748-717X-5-5 20109218 PMC2827477

[B36] Stupp R. Hegi M. E. Mason W. P. Van Den Bent M. J. Taphoorn M. J. Janzer R. C. (2009). Effects of radiotherapy with concomitant and adjuvant temozolomide versus radiotherapy alone on survival in glioblastoma in a randomised phase III study: 5-year analysis of the EORTC-NCIC trial. Lancet Oncol. 10, 459–466 10.1016/S1470-2045(09)70172-X 19269895

[B37] Thorwarth D. Eschmann S. M. Paulsen F. Alber M. (2007a). Hypoxia dose painting by numbers: a planning study. Int. J. Radiat. Oncol. Biol. Phys. 68, 291–300 10.1016/j.ijrobp.2006.11.061 17448882

[B38] Thorwarth D. Eschmann S. M. Paulsen F. Alber M. (2007b). A model of reoxygenation dynamics of head-and-neck tumors based on serial 18F-fluoromisonidazole positron emission tomography investigations. Int. J. Radiat. Oncol. Biol. Phys. 68, 515–521 10.1016/j.ijrobp.2006.11.061 17398015

[B39] Thorwarth D. Geets X. Paiusco M. (2010). Physical radiotherapy treatment planning based on functional PET/CT data. Radiother. Oncol. 96, 317–324 10.1016/j.radonc.2010.07.012 20673689

[B40] Tsai C. S. Lai C. H. Chang T. C. Yen T. C. Ng K. K. Hsueh S. (2010). A prospective randomized trial to study the impact of pretreatment FDG-PET for cervical cancer patients with MRI-detected positive pelvic but negative para-aortic lymphadenopathy. Int. J. Radiat. Oncol. Biol. Phys. 76, 477–484 10.1016/j.ijrobp.2009.02.020 19464824

[B41] Veldeman L. Madani I. Hulstaert F. De Meerleer G. Mareel M. De Neve W. (2008). Evidence behind use of intensity-modulated radiotherapy: a systematic review of comparative clinical studies. Lancet Oncol. 9, 367–375 10.1016/S1470-2045(08)70098-6 18374290

[B42] Walter F. Cloughesy T. Walter M. A. Lai A. Nghiemphu P. Wagle N. (2012). Impact of 3,4-dihydroxy-6-18F-fluoro-L-phenylalanine PET/CT on managing patients with brain tumors: the referring physician's perspective. J. Nucl. Med. 53, 393–398 10.2967/jnumed.111.095711 22323780

[B43] Weber D. C. Casanova N. Zilli T. Buchegger F. Rouzaud M. Nouet P. (2009). Recurrence pattern after [(18)F]fluoroethyltyrosine-positron emission tomography-guided radiotherapy for high-grade glioma: a prospective study. Radiother. Oncol. 93, 586–592 10.1016/j.radonc.2009.08.043 19782417

[B44] Weber D. C. Zilli T. Buchegger F. Casanova N. Haller G. Rouzaud M. (2008). [(18)F]fluoroethyltyrosine-positron emission tomography-guided radiotherapy for high-grade glioma. Radiat. Oncol. 3, 44 10.1186/1748-717X-3-44 19108742 PMC2628662

[B45] Weber W. A. (2006). Positron emission tomography as an imaging biomarker. J. Clin. Oncol. 24, 3282–3292 10.1200/JCO.2006.06.6068 16829652

[B46] Wen P. Y. Macdonald D. R. Reardon D. A. Cloughesy T. F. Sorensen A. G. Galanis E. (2010). Updated response assessment criteria for high-grade gliomas: response assessment in neuro-oncology working group. J. Clin. Oncol. 28, 1963–1972 10.1200/JCO.2009.27.6014 20231676

[B47] Wilbert J. Guckenberger M. Polat B. Sauer O. Vogele M. Flentje M. (2010). Semi-robotic 6 degree of freedom positioning for intracranial high precision radiotherapy; first phantom and clinical results. Radiat. Oncol. 5, 42 10.1186/1748-717X-5-42 20504338 PMC2890022

[B48] Wurschmidt F. Petersen C. Wahl A. Dahle J. Kretschmer M. (2011). [18F]fluoroethylcholine-PET/CT imaging for radiation treatment planning of recurrent and primary prostate cancer with dose escalation to PET/CT-positive lymph nodes. Radiat. Oncol. 6, 44 10.1186/1748-717X-6-44 21529377 PMC3095991

